# Models and regressions to describe primary damage in silicon carbide

**DOI:** 10.1038/s41598-020-67070-x

**Published:** 2020-06-26

**Authors:** G. Bonny, L. Buongiorno, A. Bakaev, N. Castin

**Affiliations:** SCK CEN, Nuclear Materials Science Institute, Boeretang 200, B-2400 Mol, Belgium

**Keywords:** Structure of solids and liquids, Ceramics, Nuclear fusion and fission

## Abstract

Silicon carbide (SiC) and SiC/SiC composites are important candidate materials for use in the nuclear industry. Coarse grain models are the only tools capable of modelling defect accumulation under different irradiation conditions at a realistic time and length scale. The core of any such model is the so-called “source term”, which is described by the primary damage. In the present work, classical molecular dynamics (MD), binary collision approximation (BCA) and NRT model are applied to describe collision cascades in 3C-SiC with primary knock-on atom (PKA) energy in the range 1–100 keV. As such, BCA and NRT are benchmarked against MD. Particular care was taken to account for electronic stopping and the use of a threshold displacement energy consistent with density functional theory and experiment. Models and regressions are developed to characterize the primary damage in terms of number of stable Frenkel pairs and their cluster size distribution, anti-sites, and defect type. As such, an accurate cascade database is developed with simple descriptors. One of the main results shows that the defect cluster size distribution follows the geometric distribution rather than a power law.

## Introduction

Silicon carbide (SiC) and SiC/SiC composites are candidate materials for use in fission and fusion applications. SiC is an attractive material due to its favorable neutronic and high-temperature properties. In fusion applications, SiC/SiC composites are considered as structural and/or functional material in the tritium breeding blanket module^1–3^. In fission applications, SiC and SiC/SiC composites are considered as cladding material, ranging from coated particle fuels to advanced accident tolerant fuel claddings in light water reactors^1,2^.

Under irradiation, the attractive properties of SiC are known to degrade as the material’s microstructure is modified. Depending on the irradiation conditions, point-defects accumulate into defect clusters that may provoke macroscopic swelling and amorphization of the material^2^.

Snead and Katoh^4^ developed a comprehensive map of the evolution of microstructural features in SiC under irradiation (both ion and neutron) with dose and irradiation temperature. Initially, damage accumulates in SiC in the form of black spot defects, i.e., defect clusters that are too small for adequate characterization. As either the temperature or dose (dpa) increases, interstitials gather to form faulted Frank loops. When both temperature and dose are increased, voids and unfaulted loops develop.

At nearly all temperatures, SiC swells, despite the lack of cavities at low temperatures and doses^4^. A comprehensive overview of the trend of swelling with temperature is given in^4^. Below a critical temperature (about 150 °C), swelling is primarily caused by amorphization of the SiC material as strain accumulates from irradiation‐induced defects from either ion- or neutron irradiation^5–8^. In the intermediate temperature regime, above the critical amorphization temperature (about 200 °C) and up to the vacancy mobility temperature (about 1000 °C), the swelling of SiC under neutron irradiation appears to saturate with irradiation dose and decreases with irradiation temperature^4^.

The works by Katoh *et al*.^5^ and Kishimoto *et al*.^9^ demonstrated the effects of helium on the swelling in SiC and SiC/SiC composites. In both works, the addition of helium resulted in up to 1.5 times larger amounts of swelling at temperatures up to 1000 °C in SiC. At temperatures higher than 1000 °C, the addition of helium has a negligible effect on the swelling.

Theoretical works employing various density functional theory (DFT) methods have been applied to determine the formation and migration energy of carbon and silicon point-defects^10–13^. Furthermore, the clustering behavior of H and He, and their interaction with point-defect clusters was also studied by DFT^14–18^. From these works, it was concluded that vacancy (clusters) have a strong impact on the stability and mobility of He and H clusters.

Conversely, the mixed experimental and theoretical works by Daghbouj *et al*.^19,20^ showed that He and H also have an important impact on the formation and stability of vacancy clusters filled with hydrogen/helium. It was found that vacancy clusters grow via Ostwald ripening (vacancy diffusion) and coalescence (because of the overlap of the strain fields around the small voids due to the pressure exerted by He or H_2_ gas) where hydrogen and helium help stabilize the vacancy clusters.

Thus, clearly, regardless the irradiation type, the initial damage distribution has an important impact on the long-term damage evolution, especially when H and/or He impurities are present.

To model defect accumulation in SiC and SiC/SiC composites at realistic time and length scales, coarse grain models are necessary. In the literature, cluster dynamics^21^ and object kinetic Monte Carlo models^22,23^ have been developed to study defect accumulation in SiC. The starting point of any such model is the so-called “source term”, i.e., primary damage.

In ceramics, the NRT model^24^ and binary collision approximation (BCA) are accepted to be good first approximations to describe primary damage^25^. This is because in ceramics, unlike metals, most defects are formed as isolated Frenkel pairs with limited clustering. However, this approach does not provide information on the type of produced Frenkel pairs, C or Si vacancy/interstitial, number/type of anti-sites, nor size distribution of vacancy/interstitial clusters. Although little clustering is expected, in the work by Liu *et al*.^21^, it was shown that a cluster dynamics model employing primary damage described as isolated Frenkel pairs was insufficient to reproduce the experimentally observed defect sizes and densities. It was argued that spatial correlations and clustering are likely necessary to obtain results consistent with experiment.

Therefore, the focus of the present work is to develop models and regressions to provide a detailed characterization of the primary damage in 3C-SiC (β-SiC). The primary damage is generated by simulating collision cascades employing classical molecular dynamics (MD), including electronic stopping, for a primary knock-on atom (PKA) energy in the wide range 1–100 keV. Although studies describing collision cascades in SiC are available in the literature^26–32^, in none of those electronic stopping is accounted for, which will be shown to play an important role.

Models and regressions are developed to characterize the primary damage in terms of number of generated stable Frenkel pairs and their cluster size distribution, anti-sites, and defect type, i.e., C/Si vacancies V_C_/V_Si_, C/Si interstitials I_C_/I_Si_ and C/Si anti-sites C_Si_/Si_C_. Where possible, an in-depth comparison is made with data from the literature as well as with the binary collision approximation (BCA)^25,33^ and the NRT model^24^. The latter two methods are computationally efficient and their validity compared to MD is assessed. Thus, the present study allows to fully characterize defect production in 3C-SiC using simple models and regressions.

The paper is organized as follows. Following this introduction, the computational details are summarized in section 2. In section 3, the stopping power is characterized such that electronic stopping is consistent with stopping and range of ions in matter (SRIM) tabulations^25,34^ and the threshold displacement energy (TDE) is consistent to density functional theory (DFT) and experimental data. In section 4, the results from our MD simulations are presented and where appropriate compared to the literature, BCA simulations and the NRT model. In section 5, an analytical model based on the geometric distribution is developed to describe the defect cluster size distribution. The paper is finalized with conclusions in section 6.

## Computational Details

Classical molecular dynamics (MD) simulations were applied using the large-scale atomic molecular massively parallel simulator (LAMMPS)^35^. To describe the interatomic interactions in 3C-SiC, the potential developed by Erhart and Albe^36^ (henceforth ERH) was used. This potential reproduces the DFT computed point defect energetics in SiC reasonably well (see also section 3), and also provides an excellent description of pure Si and graphite. This may be important for consistency with future studies considering SiC/SiC composites.

With the selected potential (ERH), cascade simulations were performed at zero Kelvin to construct a database where only ballistic effects are accounted for. As shown in the work by Samolyuk *et al*.^30^, the diffusion barriers of point-defects in SiC are not always described accurately by empirical potentials. By performing the simulations at zero Kelvin, inaccuracies on the resulting cascades due to an inaccurate description of the diffusion barriers are minimized.

Consistent with the work by Samolyuk *et al*.^30^, a variable timestep in the range 0.01–1 fs was used such that the maximum distance a particle can travel within a single step is 0.015 Å. This distance corresponds to the most probable distance travelled by a C atom during 1 fs (typical MD timestep) in a lattice equilibrated at 1600 K following the Maxwell-Boltzmann distribution.

To account for electronic stopping, a friction term consistent with the stopping and range of ions in matter (SRIM) tabulations^25^ was added. Thereby communication between atomic and electronic lattice is neglected and only the net result on the atomic lattice is accounted for. The nuclear stopping was guaranteed by the universal screened Coulomb function, so-called “ZBL function”, at short distances^25^.

Given the stiffened potential, the threshold displacement energy (TDE) for both Si and C is estimated in the $[100]$, $[110]$, $[111]$ and $[\bar{1}\bar{1}\bar{1}]$ directions at zero Kelvin. To do so, the PKA energy was varied on a 1 eV grid using ten independent runs in a given direction. For this, a box containing 1728 atoms (6 × 6 × 6 ${a}_{0}^{3}$) was used. The lowest energy in any run that generated a stable Frenkel pair is taken as TDE.

To compute the formation energy of point-defect configurations, the same box size (6 × 6 × 6 ${a}_{0}^{3}$) is used. The configurations were relaxed using the conjugate gradient method at constant volume. The formation energy, ${E}_{f}$, of defect configuration $D$ was obtained following^36^,

1 $${E}_{f}(D)=E(D)-\frac{1}{2}({n}_{{\rm{Si}}}+{n}_{{\rm{C}}}){\mu }_{{\rm{SiC}}}^{3{\rm{C}}}-\frac{1}{2}({n}_{{\rm{Si}}}-{n}_{{\rm{C}}})({\mu }_{{\rm{Si}}}^{{\rm{diam}}}-{\mu }_{{\rm{C}}}^{{\rm{grap}}}),$$

with $E(D)$ the total energy of the box containing $D$, ${n}_{i}$ the number of atoms of type $i$, ${\mu }_{{\rm{SiC}}}^{3{\rm{C}}}$ the chemical potential of 3C-SiC, ${\mu }_{{\rm{Si}}}^{{\rm{diam}}}$ the chemical potential of diamond Si and ${\mu }_{{\rm{C}}}^{{\rm{grap}}}$ the chemical potential of hexagonal graphite.

Cascades were simulated at constant equilibrium volume with a Si-PKA energy, ${E}_{PKA}^{{\rm{Si}}}$, of 1, 5, 10, 50 and 100 keV in a high index direction <135>, consistent with other works^26,27,30^. Such a direction reduces channeling effects that lead to unnecessary complications. During channeling, the PKA and recoils disperse with minimum energy loss, typically along close packed directions. As a result of the channeling, the cascade may self-interact through the periodic boundaries.

Variations of a few percent on this ideal direction were applied to allow for statistics to be collected, i.e., for each ${E}_{PKA}^{{\rm{Si}}}$, the cascade was run ten times. Simulation boxes of size 80 × 80 × 80 (4.096 × 10^6^ atoms) for ${E}_{PKA}^{{\rm{Si}}}\,=\,$1–10 keV, 100 × 100 × 100 ${a}_{0}^{3}$ (8 × 10^6^ atoms) for ${E}_{PKA}^{{\rm{Si}}}\,=$ 50 keV and 200 × 200 × 200 ${a}_{0}^{3}$ (64 × 10^6^ atoms) for ${E}_{PKA}^{{\rm{Si}}}\,=$ 100 keV were used, with ${a}_{0}=4.36$ Å the equilibrium lattice parameter following ERH. These simulation boxes proved to be large enough to avoid self-interaction of the cascade through the periodic boundaries (see also Supplementary Material). In all simulations, periodic boundary conditions were applied in three dimensions. The simulations were performed up to 10 ps, within which a stable number of Frenkel pairs were produced.

The simulation results (including TDE calculations) were post-processed to identify point defects using the Wigner-Seitz analysis as implemented in the open visualization tool (OVITO)^37,38^. As such, vacancies, interstitials and anti-sites could be identified. A cluster analysis was performed with a third nearest neighbor distance criterion.

Simulations using the binary collision approximation (BCA) were performed using the transport of ions in matter (TRIM) code^25,34^. The code was executed in “full cascade” mode, such that recoils were also followed in addition to the PKA. Cascades with ${E}_{PKA}^{{\rm{Si}}}\,=$ 0.5–500 keV were performed in bulk SiC for different TDEs. As TDE, the values obtained from the stiffened potential and DFT computed lower and upper bound^39,40^ were used.

## Stopping Power and Model Validation

**Figure 1:**
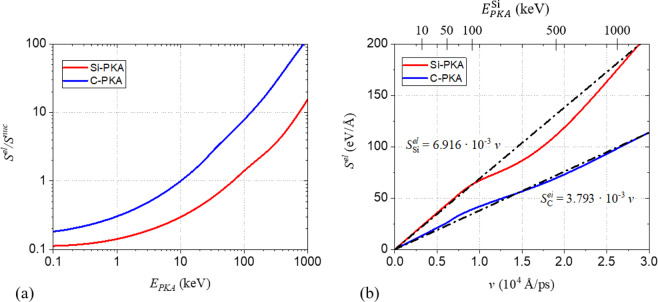
(**a**) Ratio of electronic and nuclear stopping power, ${S}^{el}/{S}^{nuc}$, as function of PKA energy, *E*_*PKA*_. (**b**) Electronic stopping power, ${S}^{el}$, as a function of particle velocity, $v$. The data is taken from SRIM stopping tables.

The stopping power of a PKA in a medium consists of the electronic stopping power, ${S}^{el}$, due to electronic excitations and nuclear stopping power, ${S}^{nuc}$, due to the Coulomb interaction with the nuclei. To illustrate the importance of both contributions, the ratio ${S}^{el}/{S}^{nuc}$ as provided by SRIM^25,34^ is plotted with *E*_*PKA*_ in Fig. 1a. As shown in the figure, ${S}^{el}\ge {S}^{nuc}$ from 10 keV and 70 keV for a C-PKA and Si-PKA, respectively. However, even for *E*_*PKA*_ as low as 0.1 keV, ${S}^{el}$ is 10–20% of ${S}^{nuc}$. Thus, for all considered cascade conditions ${S}^{el}$ is important and should be accounted for.

In the present work, ${S}^{el}$ was included by introduction of a friction term, such that the force experienced on atom *i* due to ${S}^{el}$ is proportional to its velocity, ${\overrightarrow{v}}_{i}$,

2 $${\overrightarrow{F}}_{i}=-\,\gamma \,{\overrightarrow{v}}_{i},$$

with $\gamma$ a friction coefficient fitted to ${S}^{el}$. In Fig. 1b, ${S}^{el}$ for Si and C in SiC is plotted with velocity. Clearly, Eq. 2 forms a good first approximation to describe ${S}^{el}$ (as provided in SRIM), especially for ${E}_{PKA}^{{\rm{Si}}}\le 100\,{\rm{keV}}$. Within this approximation, communication between atomic and electronic lattice is neglected and only the net result on the atomic lattice is accounted for.

The nuclear stopping power is implemented by merging the screened Coulomb (ZBL) potential by Ziegler *et al*.^25,34^, ${V}_{ZBL}$, to the equilibrium potential by Erhart and Albe^36^, ${V}_{eq}$. To ensure a smooth connection between ${V}_{ZBL}$ and ${V}_{eq}$, the scheme implemented in LAMMPS was used such that the resulting effective potential, ${V}_{eff}$, is given as,

3 $${V}_{eff}(r)=(1-{f}_{F}(r)){V}_{ZBL}(r)+{f}_{F}(r){V}_{eq}(r),$$

with

4 $${f}_{F}(r)=\frac{1}{1+\exp [-{A}_{F}(r-{r}_{C})]},$$

a Fermi-like function and the parameters ${A}_{F}=28$ Å^−1^ and ${r}_{C}=0.95$ Å. It was verified that this parameter setting did not modify the equilibrium properties of the potential (e.g. defect formation energy).

To validate the simulation set-up and used potential, in the following, point-defect properties and the threshold displacement energy (TDE) are compared to available experimental and DFT data. For later reference, values obtained from the potential by Devanath *et al*.^26,41,42^ (henceforth DEV) are also included in the comparison. In Section 4, cascade results obtained in the present work are compared to cascade results from the literature employing the DEV potential.

In Table 1, the chemical potential, formation energy of vacancies, anti-sites and various interstitial configurations obtained by the different potentials are compared to DFT values. The values for the most stable configurations are indicated in bold. For vacancies and anti-sites, both potentials reproduce the most stable DFT configurations, i.e., V_C_ and C_Si_. Quantitatively, the values obtained by the potentials are of similar magnitude as the DFT ones.

**Table 1:** Chemical potential, formation energy of vacancies, anti-sites and various interstitial configurations obtained by the different potentials compared to DFT values. The values for the most stable configurations are indicated in bold.

Config.	DFT/Exp (eV)	ERH (eV)	DEV (eV)
*Chemical potential*			
${\mu }_{{\rm{Si}}}^{{\rm{diam}}}$	−4.63^a^	−4.63	−4.63
${\mu }_{{\rm{C}}}^{{\rm{grap}}}$	−7.37^a^	−7.37	−5.31
${\mu }_{{\rm{SiC}}}^{3{\rm{C}}}$	−12.68^b^	−12.68	−12.87
*Vacancies*			
**V**_**C**_	**5.11**^**c**^**; 4.5**^**d**^	**1.91**	**5.22**
V_Si_	8.01^c^; 8.2^d^	4.56	6.77
*Anti-sites*			
**C**_**Si**_	**4.06**^**c**^**; 3.8**^**d**^	**2.18**	**0.37**
Si_C_	4.46^c^; 4.6^d^	2.26	8.06
*C-interstitials*			
C_TC_	7.78^c^; 12.4^d^	12.69	6.49
C_TSi_	7.21^c^; 10.0^d^	9.40	**3.44**
**C**^**+**^**-C** < **100** >	**4.53**^**c**^	**5.08**	9.23
C^+^-Si <100>	4.96^c^	11.02	11.15
*Si-interstitials*			
**Si**_**TC**_	**4.80**^**c**^**; 13.3**^**d**^	17.39	18.81
Si_TSi_	7.34^c^; 13.6^d^	18.11	19.49
Si^+^-C < 100>	8.68^c^	**13.16**	**13.73**
Si^+^-Si <100>	7.95^c^	17.72	18.18

^a^Experiment, ref. ^43^.

^b^Experiment, ref. ^44^.

^c^DFT data from^45^ with the corrections applied in^36^.

^d^DFT data from^46^ with the corrections applied in^36^.

For the C-interstitial, ERH predicts the C^+^-C <100> configuration as most stable configuration, consistent with DFT, while DEV predicts the C_TSi_ as most stable. For the Si-interstitial, both ERH and DEV predict the Si^+^-C < 100> configuration as most stable configuration, which is inconsistent with DFT that predicts Si_TC_ as most stable. Quantitatively, there is a large variation in magnitude between the different DFT data sets. Within this variation, the values obtained by the potentials are of similar magnitude as the DFT ones.

Thus, based on the point-defect stabilities presented in Table 1, ERH is slightly more consistent with DFT data compared to DEV. However, during high energy cascades, defect creation is expected to be dominated by ballistic effects. An important parameter for this process is the threshold displacement energy, which is discussed in the following.

The threshold displacement energy (TDE) for both Si and C is computed in the $[100]$, $[110]$, $[111]$ and $[\bar{1}\bar{1}\bar{1}]$ directions at zero Kelvin following all above settings. The TDE is essential in cascade simulations as it has a big impact on the Frenkel pair production. A comparison of the TDE computed with the present set-up with values from DFT and experiment is provided in Table 2. As shown in the table, values computed by different DFT methods vary significantly. Nevertheless, the values obtained in the present set-up are within a similar range of the DFT values. Therefore, the present set-up is considered accurate for cascade simulations.

**Table 2:** Threshold displacement energy (TDE) as obtained by experiment, density functional theory (DFT) and potentials.

Method	[100]		$[{\bf{110}}]$		[111]		$[\bar{{\bf{1}}}\bar{{\bf{1}}}\bar{{\bf{1}}}]$		Weighted Average	
	Si	C	Si	C	Si	C	Si	C	Si	C
e^-^ irr and TEM^48^	54 ± 2		54 ± 2		90 ± 2		90 ± 2		65	
DFT, Siesta code^39^	49.5	20.0	70.0	22.5	105.0	20.5	62.0	47.5	69.4	25.5
DFT, GP code^40^	46	18	45	14	22	38	21	16	38	19
ERH potential	34	19	58	38	31	31	38	58	45	36
DEV potential	36	31	71	38	113	28	39	71	64	40

For later reference, the values for the DEV potential^26,41,42^ as reported in^47^ are also included as some results will be compared to cascade results obtained with that potential. The values of that potential are higher than ERH, but still in a similar range as the DFT data.

## High Energy Cascades

**Figure 2:**
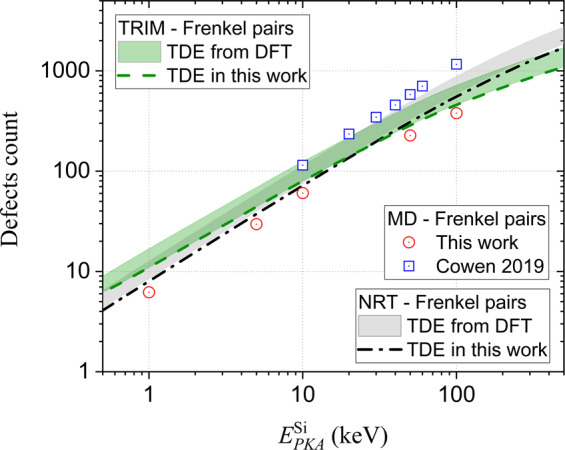
Frenkel pair count as a function of ${E}_{PKA}^{{\rm{Si}}}$ for cascades simulated by different methods. The size of the error bar on the MD data is equal to, or smaller than the size of the symbol.

The time evolution of the defect populations and animations of the simulated cascades are provided in the Supplementary Material. The results of the cascade simulations in terms of total number of stable Frenkel pairs as a function of ${E}_{PKA}^{{\rm{Si}}}$ are summarized in Fig. 2. In the same figure, estimates using TRIM simulations and the NRT model^24^ are displayed for different TDEs. The considered TDE represent weighted averages over the different directions with the limiting values predicted by DFT (shaded curves) and TDE obtained from ERH. These values allow to estimate the sensitivity of TDE on the number of produced Frenkel pairs and allow for direct comparison between TRIM, NRT and MD.

As shown in the figure, the effect of TDE is limited, i.e., less than a factor two in total number of produced Frenkel pairs. The differences between NRT, TRIM and MD are also limited, i.e., less than a factor two. The values from TRIM simulations and NRT formula are essentially equivalent to the ones from the MD simulations. For ${E}_{PKA}^{{\rm{Si}}}\le 10\,{\rm{keV}}$ the NRT model yields the closest agreement with MD, while for ${E}_{PKA}^{{\rm{Si}}}\ge 50\,{\rm{keV}}$ the TRIM simulations provide the best agreement. Thus, the total number of Frenkel pairs can be accurately estimated by computationally efficient methods like TRIM and NRT model.

For comparison, the data from the study by Cowen *et al*.^47^ are added to Fig. 2. In that work, the potential by Devanathan *et al*.^42^ is used and its TDE is summarized in Table 1. As shown in Table 1, the TDE is higher than for ERH and close to the largest values predicted by DFT. Therefore, it is striking that the number of observed Frenkel pairs is systematically higher than our MD, TRIM and NRT results. Clearly, the increased number of observed Frenkel pairs is related to the absence of electronic stopping in the simulation set-up by Cowen *et al*.^47^. This highlights the importance of accounting for electronic stopping in the simulations set-up.

**Figure 3:**
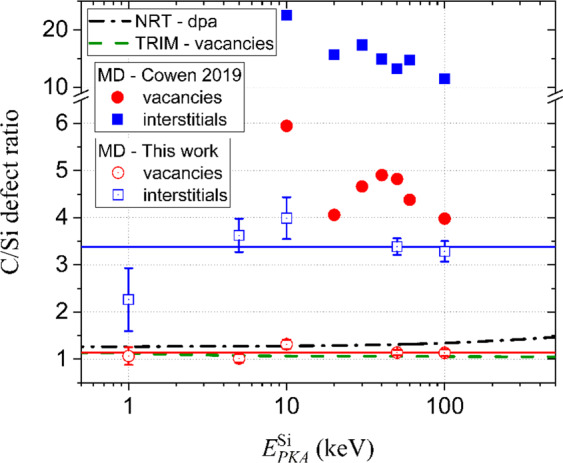
C/Si defect ratio for vacancies and interstitials as a function of ${E}_{PKA}^{{\rm{Si}}}$ computed by different methods.

In Fig. 3, the ratio of V_C_/V_Si_ and I_C_/I_Si_ obtained from MD for all investigated ${E}_{PKA}^{{\rm{Si}}}$ are summarized and where possible, compared to TRIM and the NRT model. It is noted that the large error bar for 1 keV cascades is due to the low statistics, i.e., low number of generated Frenkel pairs. It is observed that for both interstitials and vacancies the C/Si ratios remain constant for all investigated ${E}_{PKA}^{{\rm{Si}}}$. Also, for both interstitials and vacancies, more C interstitials/vacancies are produced than Si interstitials/vacancies. Ratios of V_C_/V_Si_=1.1 and I_C_/I_Si_=3.4 are observed.

The C/Si ratio derived from TRIM and NRT model is in excellent agreement with the MD results for vacancies. It is noted that the C/Si ratio for vacancies is equal to Si-TDE /C-TDE. Thus, both TRIM and NRT (or Si-TDE /C-TDE) provide an accurate estimate for V_C_/V_Si_, and based on our MD results, I_C_/I_Si_ is about three times the V_C_/V_Si_ ratio.

For comparison, the results obtained in the study by Cowen *et al*.^32^ are added to Fig. 3. In that work, the ratios I_C_/I_Si_ = 15.7 and V_C_/V_Si_ = 4.7 are at large discrepancy to the values found in the present work. Also, V_C_/V_Si_ is not equal to Si-TDE/C-TDE. However, it is striking that consistent with our results, the I_C_/I_Si_ ratio is also about three times larger than V_C_/V_Si_.

Qualitatively, for both ERH and DEV, I_C_/I_Si_ > 1 and V_C_/V_Si_ > 1 is consistent with the difference in formation energy shown in Table 1. The formation energy for the most stable I_C_ and V_C_ configurations is lower than the one for the most stable I_Si_ and V_Si_ configurations, respectively. However, a quantitative correlation could not be found. The quantitative differences are likely the result of non-trivial differences in dynamic behavior between both potentials.

**Figure 4:**
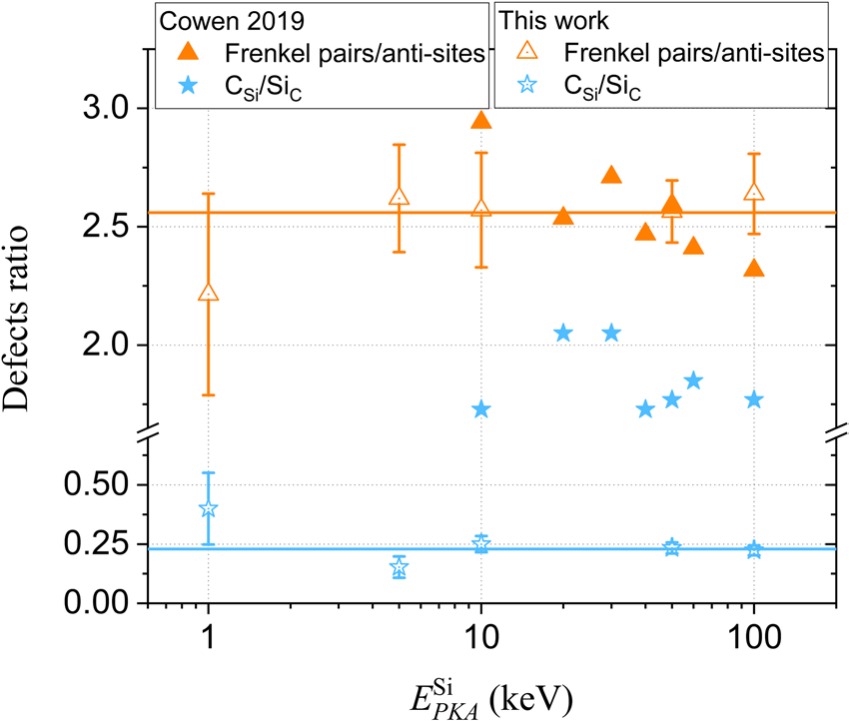
The ratio of the number of Frenkel pairs over the number of anti-sites and the ratio of C_Si_ over Si_C_ anti-sites as a function of ${E}_{PKA}^{{\rm{Si}}}$.

In Fig. 4, the ratio of Frenkel pairs/anti-sites and C_Si_/Si_C_ anti-sites for all investigated ${E}_{PKA}^{{\rm{Si}}}$ is plotted. These quantities can only be estimated from MD and appear to be constant with ${E}_{PKA}^{{\rm{Si}}}$. For any ${E}_{PKA}^{{\rm{Si}}}$, the number of anti-sites is ~2.5 times smaller than the total number of Frenkel pairs, with C_Si_/Si_C_ = 0.25. For comparison, the same ratios taken from the study of Cowen *et al*.^47^ are added to Fig. 4. While the Frenkel pairs/anti-sites ratio is in good agreement with our results, the C_Si_/Si_C_ ratio is very different. While in our case the number of Si_C_ anti-sites outnumber the C_Si_ anti-sites, the opposite is true for the results by Cowen *et al*.

The large discrepancy between the results of both potentials is reflected in their difference in formation energy of C_Si_ and Si_C_. While for ERH the formation energy of C_Si_ is similar to the one of Si_C_, for DEV the formation energy of C_Si_ is an order of magnitude lower than the one for Si_C_. A priori, the values predicted by ERH (used in this work) are more consistent with DFT than the values predicted by DEV (the work by Cowen *et al*.).

In summary, the number of produced Frenkel pairs can be described by the NRT model and TRIM simulations. Thereby accounting for electronic stopping is essential. The number of anti-sites is about a factor 2.5 lower than the total number of Frenkel pairs and the C/Si ratio for interstitials is about 3 times larger than for vacancies. These results are consistent with the work of Cowen *et al*.^32^ and therefore independent of potential. The following ratios were observed: V_C_/V_Si_ = 1.1 and C_Si_/Si_C_ = 0.25, which is at large discrepancy with the work of Cowen *et al*.^32^. The reason for these discrepancies are at least qualitatively reflected by the differences in formation energy of the respective point-defects by the different potentials used in both works.

**Figure 5:**
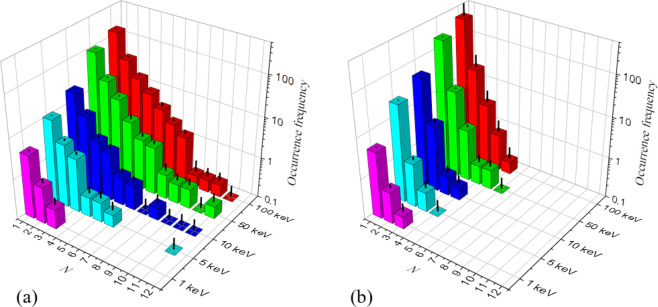
Occurrence frequency, $f(N)$, of (**a**) vacancy and (**b**) interstitial clusters of size $N$ for a given ${E}_{PKA}^{{\rm{Si}}}$. The standard error is indicated by vertical black lines.

The occurrence frequency, $f(N)$, of a defect cluster of size $N$ generated in a cascade for given ${E}_{PKA}^{{\rm{Si}}}$ is summarized in Fig. 5, for both vacancy and interstitial clusters. As expected for ceramics, defect cluster sizes are limited and decrease progressively with decreasing ${E}_{PKA}^{{\rm{Si}}}$. It is found that vacancy clusters are about twice the size of interstitial clusters. For ${E}_{PKA}^{{\rm{Si}}}=100\,{\rm{keV}}$, the largest observed vacancy cluster contains 12 vacancies, while the largest interstitial cluster contains 5 interstitials. In the next section, an analytical model to describe the defect cluster size distribution is developed.

## Analytical Model

Based on the cluster occurrence frequency, the occurrence probability, $P(N)$, of a point defect cluster of size $N$ is defined as,

5 $$P(N)=\frac{f(N)}{{\sum }_{k=1}^{{\rm{\infty }}}f(k)}.$$

**Figure 6:**
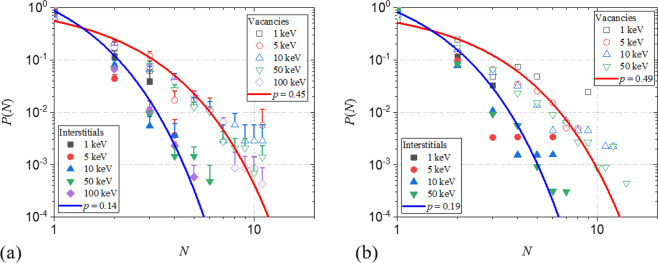
Occurrence probability, $P(N)$, of a cluster of size $N$ for vacancy and interstitial clusters for all investigated ${E}_{PKA}^{{\rm{Si}}}$ (**a**) in the present work and (**b**) from the study by Liu *et al*.^31^. The best-fit geometric distribution functions are superposed.

This is the probability of occurrence of a cluster of size $N$ amongst all observed clusters. The $P(N)$ for vacancy and interstitial clusters obtained from our cascade simulations are plotted in Fig. 6a, for all investigated ${E}_{PKA}^{{\rm{Si}}}$. Interestingly, for both vacancies and interstitials, all data scales to a single curve, independent of ${E}_{PKA}^{{\rm{Si}}}$. This indicates that the process of cluster creation occurs ballisticly, i.e. with a certain probability, $p$, without correlations. As such, a higher ${E}_{PKA}^{{\rm{Si}}}$ leads to the occurrence of larger clusters, only because the number of trials is increased, i.e. the Frenkel pair production. The clustering probability, on the other hand, remains constant. Within this scope, $P(N)$ can be described by the geometric distribution.

The geometric distribution describes the probability that the first failure (the point-defect does not cluster) requires $N$ Bernoulli trials (thus a cluster of size $N$ is obtained) for a given probability $p$ for success (clustering probability) and is given as,

6 $$P(N)={p}^{N-1}(1-p).$$

The best fit of Eq. 6 to the data for vacancies and interstitials, is obtained for *p* = 0.45 and $p=0.14$ for vacancy and interstitial clusters, respectively. These curves are added to Fig. 6a, where the data clearly follow a geometric distribution.

To illustrate the generality of this theory, it is also applied to the results obtained by Liu *et al*. (the data is generated from Fig. 3 of the respective paper)^31^ and added to Fig. 6b. As with our data, $P(N)$ for different PKA energy scale to a single curve for vacancy and interstitial clusters, respectively. Clearly, these data also satisfy the geometric distribution, even though the authors fitted a power law to their data. It is observed that the independent cluster probability, $p$, although slightly increased, is in the same range as found for our data. Small differences are to be expected due to differences in simulation set-up, the most important ones being: different interatomic potential and lack of electronic stopping power.

Thus, for 3C-SiC (and likely ceramics) the cluster occurrence probability is described by the geometric distribution, rather than a power law, that is adequate to describe defect clusters in tungsten^49^. The presented distribution function allows to generate cascade libraries beyond the scope of MD simulations by accounting for rare events that are inaccessible on the MD length scale.

It is emphasized that given the generality of the theory, the applicability of the geometric size distribution can probably extend beyond the case of SiC, possibly extending to a wide class of ceramics.

## Conclusions

Primary damage in 3C-SiC was characterized using classical molecular dynamics (MD), binary collision approximation (BCA) and NRT model for a PKA energy range 1–100 keV. As such, BCA and NRT are benchmarked against MD. Particular care was taken to account for electronic stopping and the use of a threshold displacement energy (TDE) consistent with density functional theory (DFT) and experiment.

It was found that the Frenkel pair production described by BCA and NRT are equivalent with an MD description. The effect of TDE, within the range provided by DFT, affects the total Frenkel pair production by less than a factor two.

The ratio of number of Frenkel pairs over number of anti-sites is about 2.5, C_Si_/Si_C_ = 0.25, V_C_/V_Si_ = 1.1 and I_C_/I_Si_ = 3.4. It is emphasized that these ratios are constant over the range of investigated PKA energy.

The defect cluster size occurrence probability was found to follow the geometric distribution rather than the power law proposed by Sand *et al*.^49^ to describe defect clusters in tungsten. The geometric distribution is characterized by the number of trials *N*, before first failure, provided each trial is a Bernoulli trial. This view is consistent with ballistic effects that dominate cascade formation in ceramics and 3C-SiC in particular. It is thus important to emphasize that the applicability of the geometric size distribution can probably extend beyond the case of SiC, possibly extending to a wide class of ceramics.

Thus, the present study allows to fully characterize defect production in 3C-SiC using simple models and regressions.

To conclude, a call is launched for experimental validation by *in-situ* transmission electron microscopy (TEM) under ion irradiation at cryogenic temperatures. Cryogenic temperature may avoid thermally driven recombination and only allow for ballistic effects to occur. If not quantitatively, the form of the distribution function can be verified.

## Supplementary inforamtion

Supplementary information.

## References

[1] Katoh Y, Snead LL (2019). Silicon carbide and its composites for nuclear applications – Historical overview. Journal of Nuclear Materials.

[2] Koyanagi T (2018). Recent progress in the development of SiC composites for nuclear fusion applications. Journal of Nuclear Materials.

[3] Snead LL (2011). Silicon carbide composites as fusion power reactor structural materials. Journal of Nuclear Materials.

[4] Snead LL (2007). Handbook of SiC properties for fuel performance modeling. Journal of Nuclear Materials.

[5] Katoh Y, Kishimoto H, Kohyama A (2002). The influences of irradiation temperature and helium production on the dimensional stability of silicon carbide. Journal of Nuclear Materials.

[6] Katoh Y, Kishimoto H, Kohyama A (2002). Low Temperature Swelling in Beta-SiC Associated with Point Defect Accumulation. MATERIALS TRANSACTIONS.

[7] Snead LL, Zinkle SJ, Hay JC, Osborne MC (1998). Amorphization of SiC under ion and neutron irradiation. Nuclear Instruments and Methods in Physics Research Section B: Beam Interactions with Materials and Atoms.

[8] Snead LL, Zinkle SJ (2002). Structural relaxation in amorphous silicon carbide. Nuclear Instruments and Methods in Physics Research Section B: Beam Interactions with Materials and Atoms.

[9] Kishimoto H, Ozawa K, Kondo S, Kohyama A (2005). Effects of Dual-Ion Irradiation on the Swelling of SiC/SiC Composites. MATERIALS TRANSACTIONS.

[10] Gao F, Weber WJ, Xiao HY, Zu XT (2009). Formation and properties of defects and small vacancy clusters in SiC: Ab initio calculations. Nuclear Instruments and Methods in Physics Research Section B: Beam Interactions with Materials and Atoms.

[11] Bockstedte M, Mattausch A, Pankratov O (2004). Ab initio study of the annealing of vacancies and interstitials in cubic SiC: Vacancy-interstitial recombination and aggregation of carbon interstitials. Physical Review B.

[12] Zheng M-J, Swaminathan N, Morgan D, Szlufarska I (2013). Energy barriers for point-defect reactions in $3C$-SiC. Physical Review B.

[13] Liao T, Roma G, Wang J (2009). First-principles study of neutral silicon interstitials in 3C- and 4H-SiC. Philosophical Magazine.

[14] Sun J (2017). The effect of irradiation-induced point defects on energetics and kinetics of hydrogen in 3C-SiC in a fusion environment. Nuclear Fusion.

[15] Zhang L, Zhang Y, Lu G-H (2009). First-principles investigation of site preference and bonding properties of neutral H in 3C–SiC. Nuclear Instruments and Methods in Physics Research Section B: Beam Interactions with Materials and Atoms.

[16] Aradi B (2001). Ab initio density-functional supercell calculations of hydrogen defects in cubic SiC. Physical Review B.

[17] Deák P (2000). Vacancies and their Complexes with H in SiC. Materials Science Forum.

[18] Zhao S, Ran G, Li F, Deng H, Gao F (2019). Ab initio study of interstitial helium clusters in 3C-SiC. Journal of Nuclear Materials.

[19] Daghbouj N (2019). Microstructural evolution of helium-irradiated 6H–SiC subjected to different irradiation conditions and annealing temperatures: A multiple characterization study. Acta Mater..

[20] Daghbouj N (2020). The structural evolution of light-ion implanted 6H-SiC single crystal: Comparison of the effect of helium and hydrogen. Acta Mater..

[21] Liu C (2017). Evolution of small defect clusters in ion-irradiated 3C-SiC: Combined cluster dynamics modeling and experimental study. Acta Mater..

[22] Watanabe Y, Morishita K, Yamamoto Y (2011). Nucleation and growth of self-interstitial atom clusters in β-SiC during irradiation: Kinetic Monte-Carlo modeling. Nuclear Instruments and Methods in Physics Research Section B: Beam Interactions with Materials and Atoms.

[23] Guo, D., Martin-Bragado, I., He, C., Zang, H. & Zhang, P. Modeling of long-term defect evolution in heavy-ion irradiated 3C-SiC: Mechanism for thermal annealing and influences of spatial correlation. **116**, 204901, 10.1063/1.4902145 (2014).

[24] Norgett MJ, Robinson MT, Torrens IM (1975). A proposed method of calculating displacement dose rates. Nuclear Engineering and Design.

[25] Ziegler, J. F., Biersack, J. P. & Littmark, U. *The stopping and range of ions in solids*. Vol. 1 (Pergamon Press (1985).

[26] Devanathan R, Weber WJ, Diaz de la Rubia T (1998). Computer simulation of a 10 keV Si displacement cascade in SiC. Nuclear Instruments and Methods in Physics Research Section B: Beam Interactions with Materials and Atoms.

[27] Gao F, Weber WJ (2000). Atomic-scale simulation of 50 keV Si displacement cascades in \ensuremath{\beta}-SiC. Physical Review B.

[28] Devanathan R, Weber WJ, Gao F (2001). Atomic scale simulation of defect production in irradiated 3C-SiC. Journal of Applied Physics.

[29] Gao F, Weber WJ, Devanathan R (2001). Atomic-scale simulation of displacement cascades and amorphization in β-SiC. Nuclear Instruments and Methods in Physics Research Section B: Beam Interactions with Materials and Atoms.

[30] Samolyuk GD, Osetsky YN, Stoller RE (2015). Molecular dynamics modeling of atomic displacement cascades in 3C–SiC: Comparison of interatomic potentials. Journal of Nuclear Materials.

[31] Liu C, Szlufarska I (2018). Distribution of defect clusters in the primary damage of ion irradiated 3C-SiC. Journal of Nuclear Materials.

[32] Cowen BJ, El-Genk MS, Hattar K, Briggs SA (2019). Investigations of irradiation effects in crystalline and amorphous SiC. Journal of Applied Physics.

[33] Biersack JP, Haggmark LG (1980). A Monte Carlo computer program for the transport of energetic ions in amorphous targets. Nuclear Instruments and Methods.

[34] Biersack JP, Ziegler JF (1982). Refined universal potentials in atomic collisions. Nuclear Instruments and Methods in Physics Research.

[35] Plimpton S (1995). Fast parallel algorithms for short-range molecular dynamics. Journal of Computational Physics.

[36] Erhart P, Albe K (2005). Analytical potential for atomistic simulations of silicon, carbon, and silicon carbide. Physical Review B.

[37] Stukowski A (2010). Visualization and analysis of atomistic simulation data with OVITO-the Open Visualization Tool. Modelling and Simulation in Materials Science and Engineering.

[38] Stukowski A (2012). Structure identification methods for atomistic simulations of crystalline materials. Modelling and Simulation in Materials Science and Engineering.

[39] Gao F, Xiao HY, Weber WJ (2011). Ab initio molecular dynamics simulations of low energy recoil events in ceramics. Nuclear Instruments and Methods in Physics Research Section B: Beam Interactions with Materials and Atoms.

[40] Lucas G, Pizzagalli L (2005). Ab initio molecular dynamics calculations of threshold displacement energies in silicon carbide. Physical Review B.

[41] Devanathan R, Diaz de la Rubia T, Weber WJ (1998). Displacement threshold energies in β-SiC. Journal of Nuclear Materials.

[42] Devanathan R, Weber WJ (2000). Displacement energy surface in 3C and 6H SiC. Journal of Nuclear Materials.

[43] Kittel, C. *Introduction to Solid State Physics* (1996).

[44] Lambrecht WRL, Segall B, Methfessel M, van Schilfgaarde M (1991). Calculated elastic constants and deformation potentials of cubic SiC. Physical Review B.

[45] Gao F, Bylaska EJ, Weber WJ, Corrales LR (2001). Ab initio and empirical-potential studies of defect properties in $3C\ensuremath{-}\mathrm{SiC}$. Physical Review B.

[46] Wang C, Bernholc J, Davis RF (1988). Formation energies, abundances, and the electronic structure of native defects in cubic SiC. Physical Review B.

[47] Cowen BJ, El-Genk MS (2018). Point defects production and energy thresholds for displacements in crystalline and amorphous SiC. Computational Materials Science.

[48] Hønstvet IA, Smallman RE, Marquis PM (1980). A determination of the atomic displacement energy in cubic silicon carbide. Philosophical Magazine A.

[49] Sand AE, Dudarev SL, Nordlund K (2013). High-energy collision cascades in tungsten: Dislocation loops structure and clustering scaling laws. EPL.

