# Supplementary material for: Models and regressions to describe primary damage in silicon carbide
Source: Sci Rep. 2020 Jun 26;10:10483. doi: 10.1038/s41598-020-67070-x (PMC7320178; doi:10.1038/s41598-020-67070-x)
Supplement: Supplementary file 1 — Supplementary information. [file 41598_2020_67070_MOESM1_ESM.zip › Supplementary_Information/10keV/evolution.htm]

10 keV


Return

|  |
| --- |
| **10 keV**    **– IC;  – ISi;  – VC;  – VSi;  – CSi;  – SiC** |
|  |
|  |

 

 

Return
