# Supplementary material for: Models and regressions to describe primary damage in silicon carbide
Source: Sci Rep. 2020 Jun 26;10:10483. doi: 10.1038/s41598-020-67070-x (PMC7320178; doi:10.1038/s41598-020-67070-x)
Supplement: Supplementary file 1 — Supplementary information. [file 41598_2020_67070_MOESM1_ESM.zip › Supplementary_Information/index.htm]

Supplementary Information


**Supplementary
Information**

**Models
and regressions to describe primary damage in silicon carbide**

 

G.
Bonny, L. Buongiorno, A. Bakaev, N. Castin

 

*SCK
CEN, Nuclear Materials Science Institute, Boeretang 200, B-2400 Mol, Belgium*

 

**List
of Abbreviations**

 

|  |  |
| --- | --- |
| IC | – Carbon interstitial |
| ISi | – Silicon interstitial |
| VC | – Carbon vacancy |
| VSi | – Silicon vacancy |
| CSi | – Carbon anti-site |
| SiC | – Silicon anti-site |
| PKA | – Primary Knock-on Atom |

 

**Details
of the Cascades**

 

In **Table 1**, the evolution of the number of point-defects
as a function of simulation time and final cascade debris for the investigated
PKA energy is shown. Each row in **Table 1** is linked to an animated gif
visualizing the evolution of the cascade debris (html version only).It is noted that a
single representative example per PKA energy was selected. The data presented in the
main manuscript is average over 10 independent runs per PKA energy.

 

**Table 1
– Evolution of the number of point-defects as a function of simulation time and
final cascade debris for the investigated PKA energy. Each row is linked to an
animated gif visualizing the evolution of the cascade debris (html version
only).  – IC;
 – ISi;  – VC;  – VSi;  – CSi;  – SiC.**

|  |  |  |
| --- | --- | --- |
| PKA energy | Defect evolution | Final configuration |
| 1 keV |  |  |
| 5 keV |  |  |
| 10 keV |  |  |
| 50 keV |  |  |
| 100 keV |  |  |
