# Supplementary material for: Models and regressions to describe primary damage in silicon carbide
Source: Sci Rep. 2020 Jun 26;10:10483. doi: 10.1038/s41598-020-67070-x (PMC7320178; doi:10.1038/s41598-020-67070-x)

Supplementary Information

Models and regressions to describe primary damage in silicon carbide

G. Bonny, L. Buongiorno, A. Bakaev, N. Castin

SCK CEN, Nuclear Materials Science Institute, Boeretang 200, B-2400 Mol, Belgium

List of Abbreviations

I_C – Carbon interstitial
I_{Si} – Silicon interstitial
V_C – Carbon vacancy
V_{Si} – Silicon vacancy
C_{Si} – Carbon anti-site
Si_C – Silicon anti-site
PKA – Primary Knock-on Atom

Details of the Cascades

In **Table 1**, the evolution of the number of point-defects as a function of simulation time and final cascade debris for the investigated PKA energy is shown. Each row in **Table 1** is linked to an animated gif visualizing the evolution of the cascade debris (html version only). It is noted that a single representative example per PKA energy was selected. The data presented in the main manuscript is average over 10 independent runs per PKA energy.

Table 1 – Evolution of the number of point-defects as a function of simulation time and final cascade debris for the investigated PKA energy. Each row is linked to an animated gif visualizing the evolution of the cascade debris (html version only). ● – I_C; ● – I_{Si}; ● – V_C; ● – V_{Si}; ● – C_{Si}; ● – Si_C.

PKA energy	Defect evolution	Final configuration
1 keV	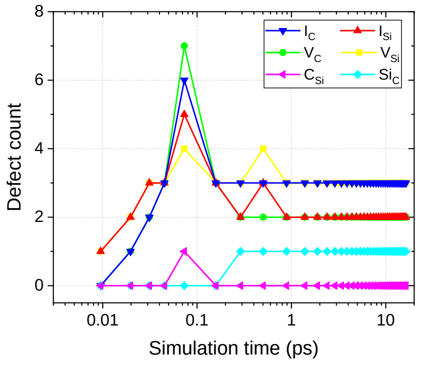	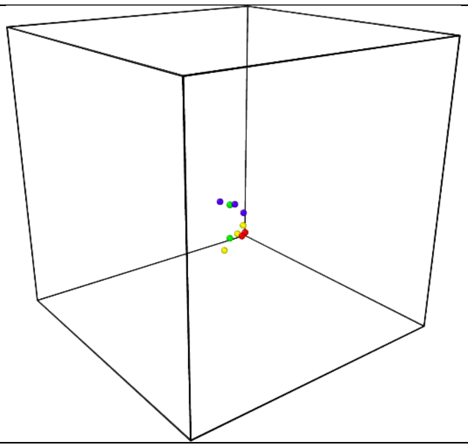

5 keV

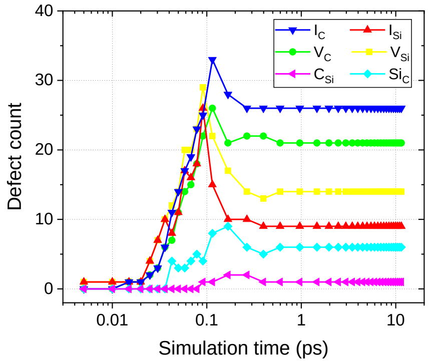

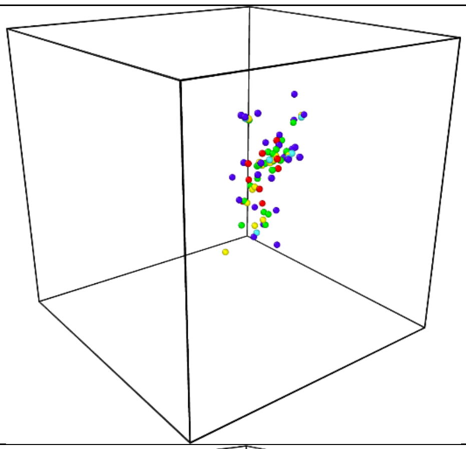

10 keV

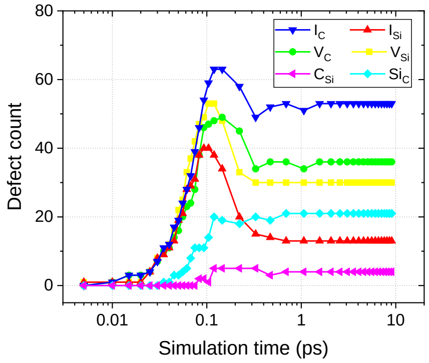

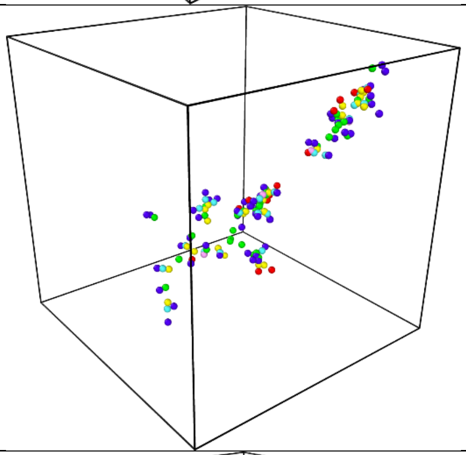

50 keV

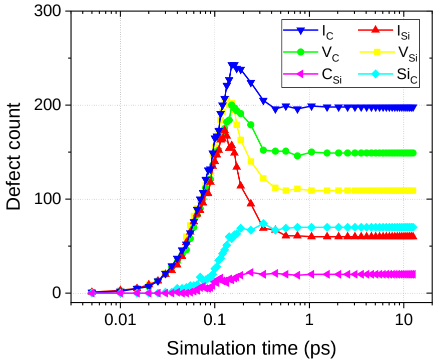

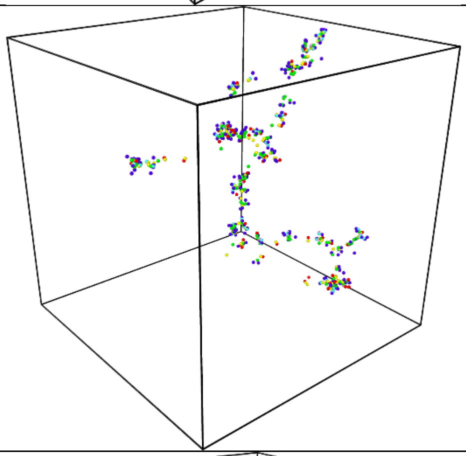

100 keV

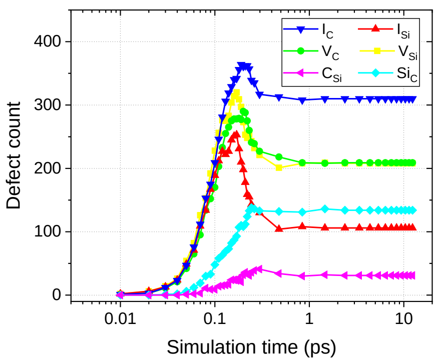

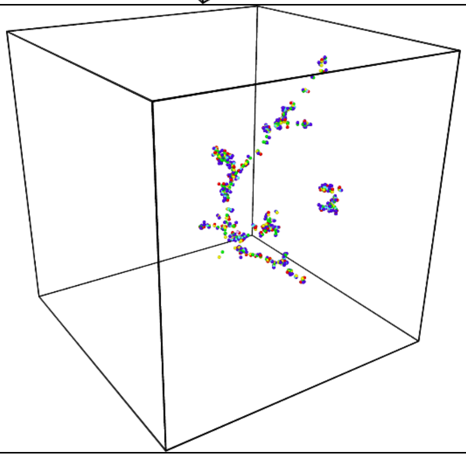

Supplement: Supplementary file 1 — Supplementary information. [file 41598_2020_67070_MOESM1_ESM.zip › Supplementary_Information/Supplementary_Information.pdf]
