# Supplementary figures and images for: Models and regressions to describe primary damage in silicon carbide
Source: Sci Rep. 2020 Jun 26;10:10483. doi: 10.1038/s41598-020-67070-x (PMC7320178; doi:10.1038/s41598-020-67070-x)

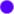

Supplement: Supplementary file 1 — Supplementary information. [file 41598_2020_67070_MOESM1_ESM.zip › Supplementary_Information/100keV/evolution_files/image001.png]

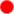

Supplement: Supplementary file 1 — Supplementary information. [file 41598_2020_67070_MOESM1_ESM.zip › Supplementary_Information/100keV/evolution_files/image002.png]

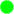

Supplement: Supplementary file 1 — Supplementary information. [file 41598_2020_67070_MOESM1_ESM.zip › Supplementary_Information/100keV/evolution_files/image003.png]

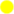

Supplement: Supplementary file 1 — Supplementary information. [file 41598_2020_67070_MOESM1_ESM.zip › Supplementary_Information/100keV/evolution_files/image004.png]

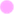

Supplement: Supplementary file 1 — Supplementary information. [file 41598_2020_67070_MOESM1_ESM.zip › Supplementary_Information/100keV/evolution_files/image005.png]

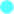

Supplement: Supplementary file 1 — Supplementary information. [file 41598_2020_67070_MOESM1_ESM.zip › Supplementary_Information/100keV/evolution_files/image006.png]

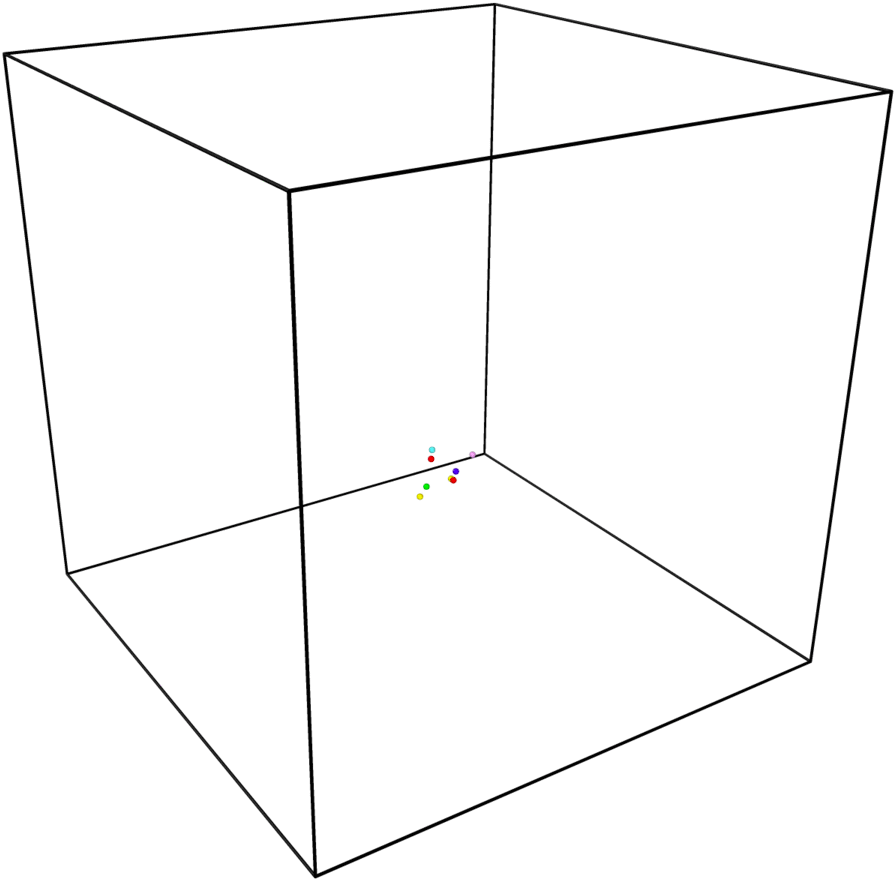

Supplement: Supplementary file 1 — Supplementary information. [file 41598_2020_67070_MOESM1_ESM.zip › Supplementary_Information/100keV/evolution_files/image007.gif]

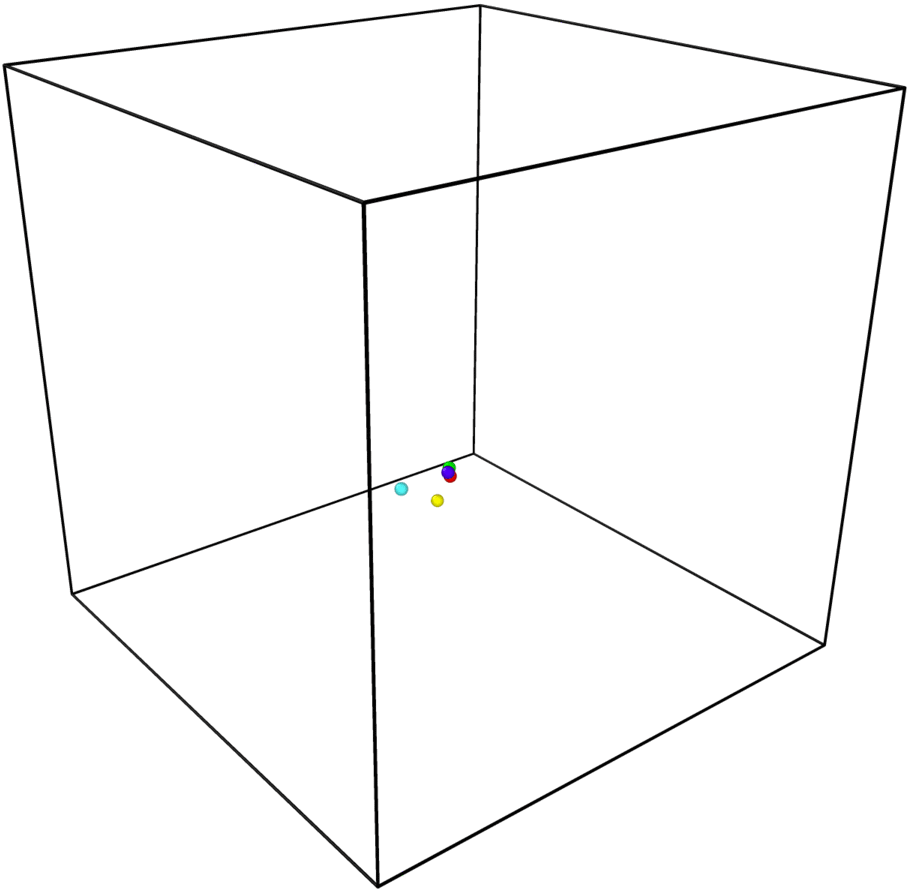

Supplement: Supplementary file 1 — Supplementary information. [file 41598_2020_67070_MOESM1_ESM.zip › Supplementary_Information/10keV/evolution_files/image007.gif]

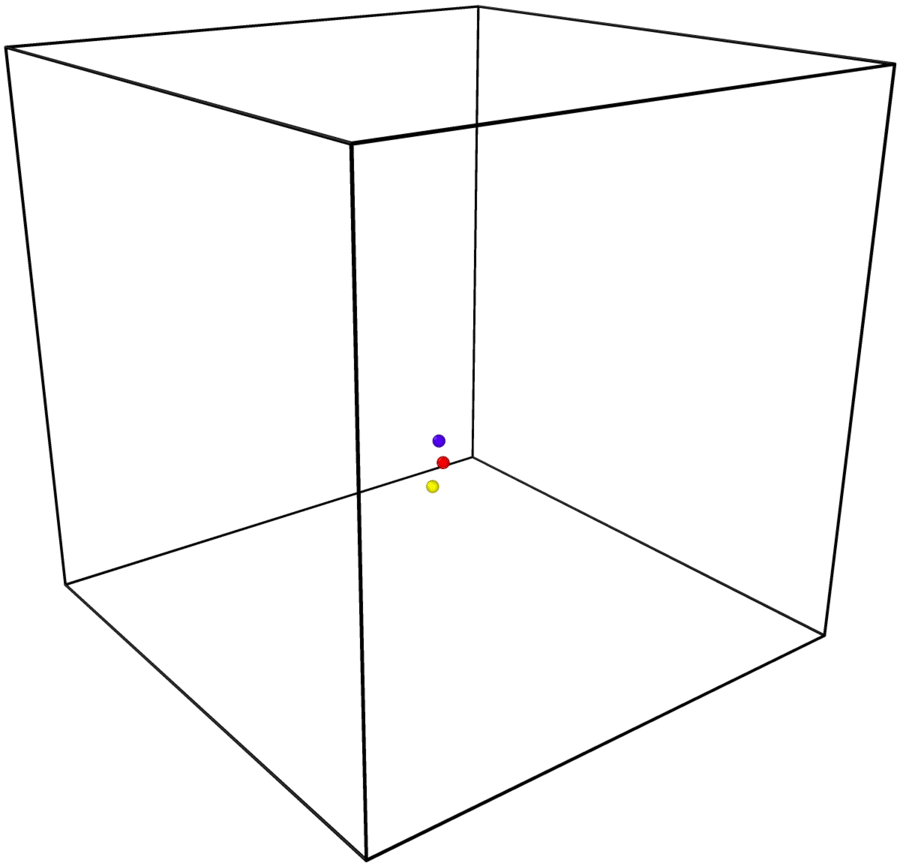

Supplement: Supplementary file 1 — Supplementary information. [file 41598_2020_67070_MOESM1_ESM.zip › Supplementary_Information/1keV/evolution_files/image007.gif]

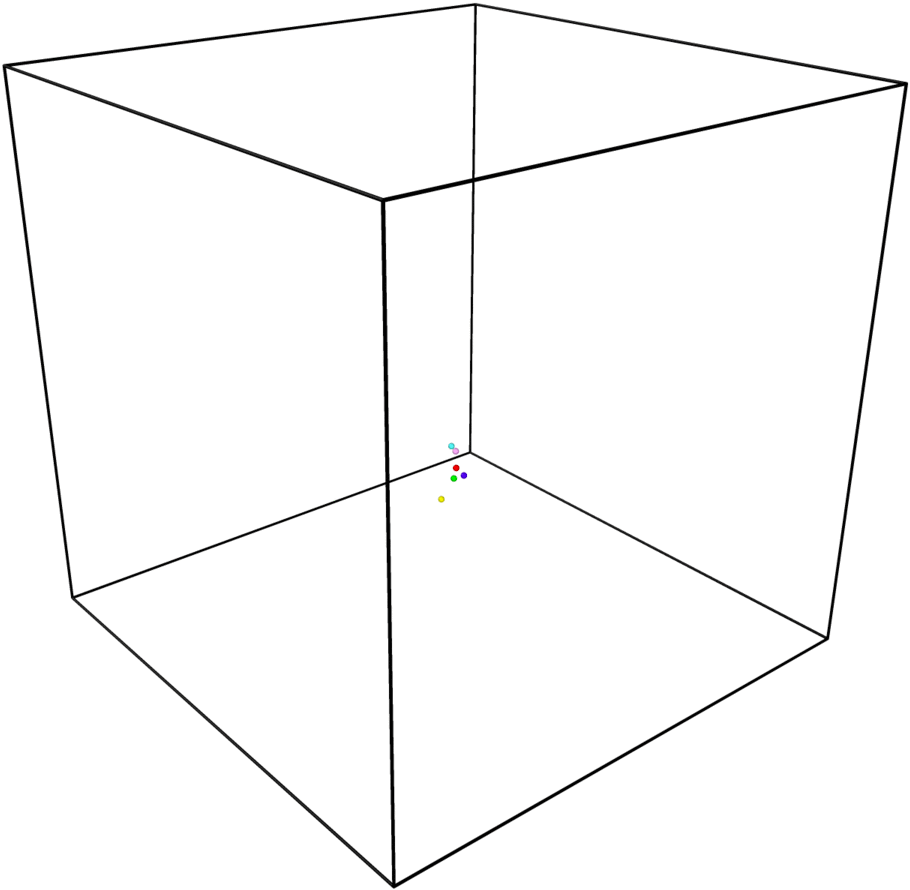

Supplement: Supplementary file 1 — Supplementary information. [file 41598_2020_67070_MOESM1_ESM.zip › Supplementary_Information/50keV/evolution_files/image007.gif]

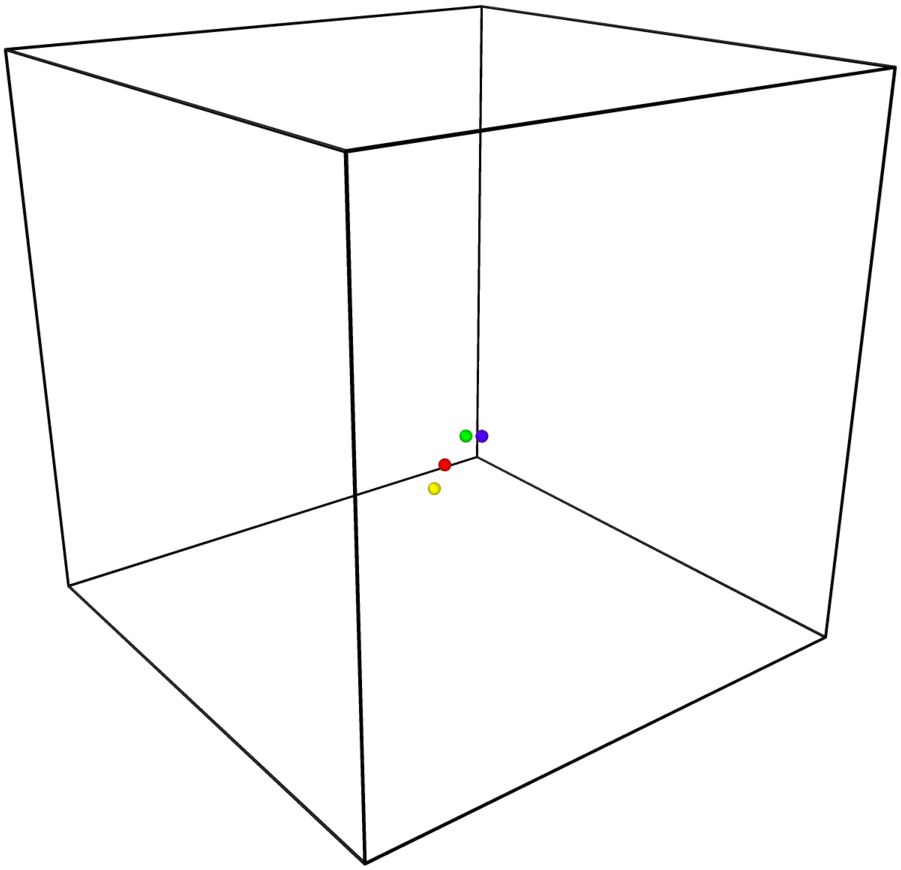

Supplement: Supplementary file 1 — Supplementary information. [file 41598_2020_67070_MOESM1_ESM.zip › Supplementary_Information/5keV/evolution_files/image007.gif]

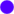

Supplement: Supplementary file 1 — Supplementary information. [file 41598_2020_67070_MOESM1_ESM.zip › Supplementary_Information/index/img001.png]

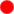

Supplement: Supplementary file 1 — Supplementary information. [file 41598_2020_67070_MOESM1_ESM.zip › Supplementary_Information/index/img002.png]

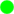

Supplement: Supplementary file 1 — Supplementary information. [file 41598_2020_67070_MOESM1_ESM.zip › Supplementary_Information/index/img003.png]

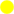

Supplement: Supplementary file 1 — Supplementary information. [file 41598_2020_67070_MOESM1_ESM.zip › Supplementary_Information/index/img004.png]

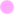

Supplement: Supplementary file 1 — Supplementary information. [file 41598_2020_67070_MOESM1_ESM.zip › Supplementary_Information/index/img005.png]

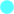

Supplement: Supplementary file 1 — Supplementary information. [file 41598_2020_67070_MOESM1_ESM.zip › Supplementary_Information/index/img006.png]

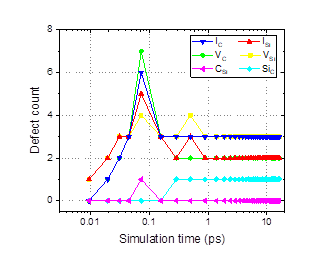

Supplement: Supplementary file 1 — Supplementary information. [file 41598_2020_67070_MOESM1_ESM.zip › Supplementary_Information/index/img007.png]

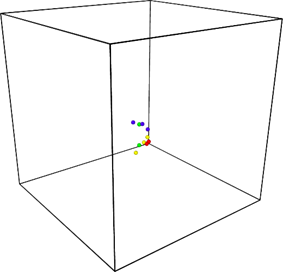

Supplement: Supplementary file 1 — Supplementary information. [file 41598_2020_67070_MOESM1_ESM.zip › Supplementary_Information/index/img008.png]

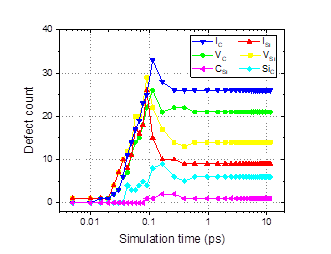

Supplement: Supplementary file 1 — Supplementary information. [file 41598_2020_67070_MOESM1_ESM.zip › Supplementary_Information/index/img009.png]

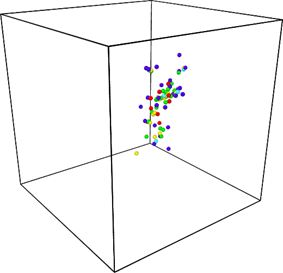

Supplement: Supplementary file 1 — Supplementary information. [file 41598_2020_67070_MOESM1_ESM.zip › Supplementary_Information/index/img010.png]

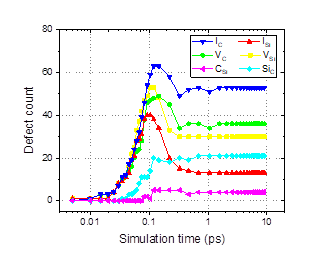

Supplement: Supplementary file 1 — Supplementary information. [file 41598_2020_67070_MOESM1_ESM.zip › Supplementary_Information/index/img011.png]

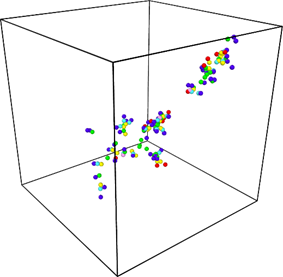

Supplement: Supplementary file 1 — Supplementary information. [file 41598_2020_67070_MOESM1_ESM.zip › Supplementary_Information/index/img012.png]

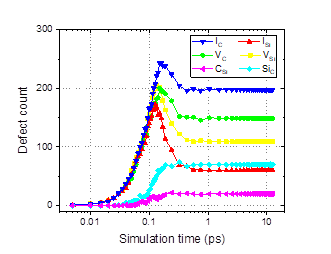

Supplement: Supplementary file 1 — Supplementary information. [file 41598_2020_67070_MOESM1_ESM.zip › Supplementary_Information/index/img013.png]

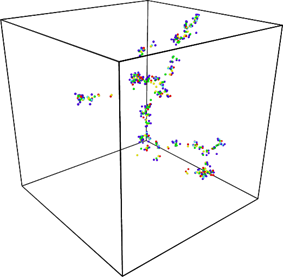

Supplement: Supplementary file 1 — Supplementary information. [file 41598_2020_67070_MOESM1_ESM.zip › Supplementary_Information/index/img014.png]

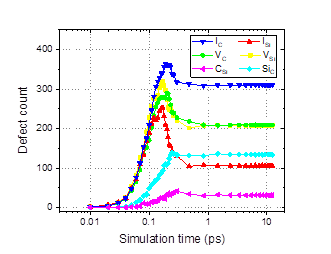

Supplement: Supplementary file 1 — Supplementary information. [file 41598_2020_67070_MOESM1_ESM.zip › Supplementary_Information/index/img015.png]

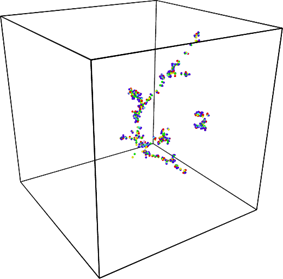

Supplement: Supplementary file 1 — Supplementary information. [file 41598_2020_67070_MOESM1_ESM.zip › Supplementary_Information/index/img016.png]

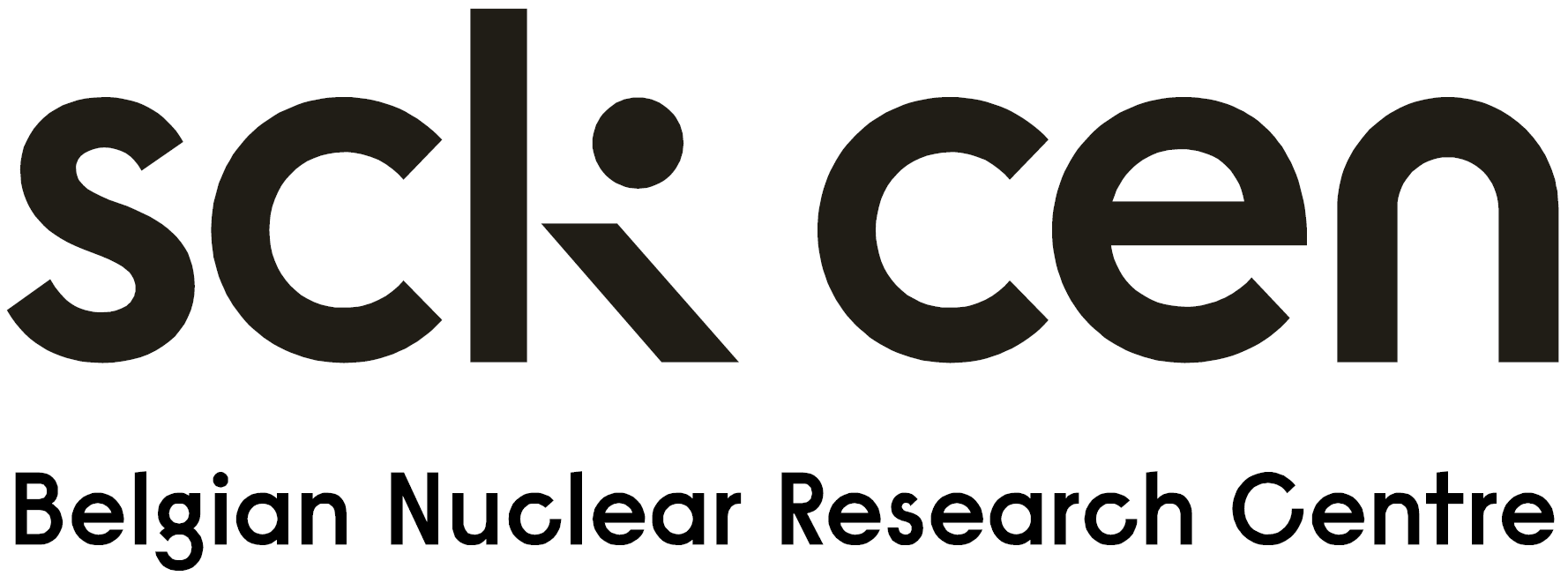

Supplement: Supplementary file 1 — Supplementary information. [file 41598_2020_67070_MOESM1_ESM.zip › Supplementary_Information/index/SCK-CEN.png]
